# Coronavirus Disease 2019 Infection and Early Diagnosis of Macrophage Activation Syndrome: A Case Report of a 62-year-old man

**DOI:** 10.1590/0037-8682-0377-2021

**Published:** 2022-03-14

**Authors:** Filipe Jonas Federico da Cruz, Eduardo Andrada Pessoa de Figueiredo, Filipe Prohaska Batista, Marcos Antônio Cavalcanti Gallindo, Andesson Carlos da Silva Fernandes

**Affiliations:** 1 Universidade Federal de Pernambuco, Hospital das Clínicas, Departamento de Geriatria, Recife, PE, Brasil.; 2 Hospital Santa Joana Recife, Departamento de Doenças Infecciosas, Recife, PE, Brasil.; 3 Hospital Santa Joana Recife, Unidade de Terapia Intensiva, Recife, PE, Brasil.

**Keywords:** Covid-19, Macrophage activation, Early diagnosis

## Abstract

A 62-year-old man presented with a history of fever, headache, anosmia, ageusia, and diarrhea for 9 days. A clinical and epidemiological diagnosis of infection with the novel coronavirus was made. After symptom refractoriness, the second step involves using human intravenous immunoglobulin. Early diagnosis of macrophage activation syndrome (MAS) involves observation of the refractory nature of clinical support treatment associated with biochemical changes to the patient's baseline characteristics, suggesting the relevance of a favorable clinical outcome of weaning from artificial life support when there is an early suspicion of a diagnosis of MAS secondary to coronavirus disease 2019 infection.

## INTRODUCTION

The 2019-2020 coronavirus pandemic has affected all over the globe. SARS-Cov-2 (severe acute respiratory syndrome coronavirus-2) is the causative agent of coronavirus disease 2019 (COVID-19), sprayed in Wuhan, China, in December 2019. It causes human infection of several tissues in the respiratory, cardiovascular, and neurological systems^1^. The severe form of the novel coronavirus infection shares clinical and laboratory characteristics with the already known hyperinflammatory^2^ and hyperferritinemic^3^ syndromes, including macrophage activation syndrome (MAS). MAS is a state of systemic hyperinflammation typified by upregulated expression of pro-inflammatory cytokines, often observed in patients with infections, malignancy, and rheumatological diseases, such as systemic juvenile idiopathic arthritis^4^. Hyperferritinemia reflects a state of inflammation in the body that actively contributes to the progression of the inflammatory process. As for the role of reflecting a state of inflammation, hyperferritinemia signals damage to the iron storage cells (hepatocytes) and the consequent release of this free ion into the circulation, resulting in endothelial injury due to oxidative stress and formation of microthrombi. It also signals macrophage hyperactivation^3^. There is biological plausibility in the literature review results regarding altered laboratory parameters at the beginning of MAS in this pathophysiological context. The findings of early drop in platelet/leukocyte count and early elevation of ferritin, lactic dehydrogenase (LDH), and transaminases can be cited^5^.

This is a case regarding the therapeutic approach and clinical outcome in a patient with severe COVID-19 infection, in which the hypothesis of MAS was considered early.

## CASE REPORT

A 62-year-old man with hypertension and chronic kidney disease was admitted to the emergency department complaining of asthenia, weight loss, anosmia, and ageusia for approximately 9 days. Symptoms were associated with frontal headaches, nasal congestion, and diarrhea. The day before admission, he had a fever episode. 

**FIGURE 1:**
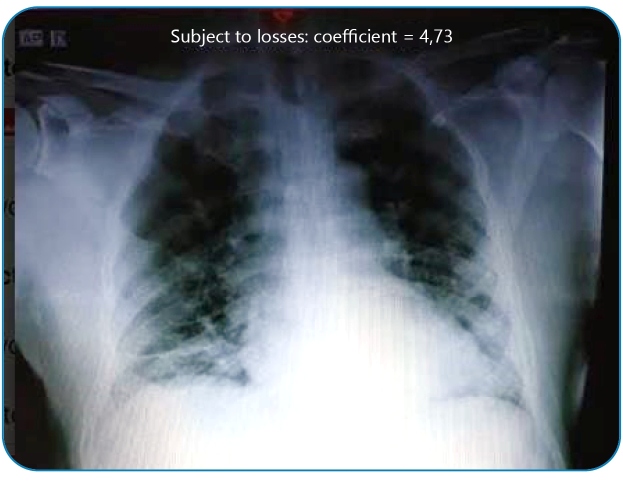
Chest radiography on the 10th day of symptoms.

The patient was conscious, oriented, and dyspneic in a prone position when he arrived at the emergency room. Chest radiography revealed bilateral interstitial infiltrates (Figure 1). The patient fulfilled the clinical criteria for the diagnosis of COVID-19 (fever, respiratory symptoms, anosmia, ageusia, and severe acute respiratory syndrome (SARS]) without any other likely cause). The first doses of hydroxychloroquine, azithromycin, and nitazoxanide were introduced based on scientific discussions at the beginning of the pandemic. The patient was admitted to the ward with supplemental oxygen and prone protocol.

On the 10^th^ day of symptoms, he experienced worsening hypoxemia and volume-refractory hypotension, which may be associated with hydroxychloroquine-induced diarrhea. The patient was referred to the intensive care unit (ICU). Antibiotic therapy with intravenous (IV) ceftaroline was initiated for bacterial pneumonia. Tocilizumab was administered following the hospital protocol based on the hypothesis of a cytokine storm associated with COVID-19.

On the 12^th^ day of symptoms, the patient developed clinical and radiological worsening and required orotracheal intubation (OTI). There was an increase in nitrogenous wastes: creatinine (Cr) 1.28 > 1.65, and rhabdomyolysis - creatine phosphokinase (CPK) 4,187. Antimicrobial therapy was expanded with intravenous meropenem, and full anticoagulation was initiated by D-dimer elevation. Due to subfebrile, radiological worsening, and hypersecretion, IV teicoplanin was started (based on a previous blood culture with coagulase-negative Staphylococcus; other cultures, including fungal cultures, were negative). Due to persistent febrile episodes, another extension of the antibiotic regimen was performed with a double dose of meropenem and the addition of polymyxin B on the 18^th^ day to cover gram-negative in-hospital germs.

On the 20^th^ day of symptoms, the patient still had persistent fever and worsening inflammatory condition; thus, corticotherapy was started with methylprednisolone succinate for 5 days (125 mg twice a day [BID] on the first day and 40 mg BID on the next 4 days). On the 23^rd^ day of symptoms, he was extubated with a non-rebreathing mask for support.

The patient experienced difficulty weaning from dexmedetomidine due to refractory delirium secondary to a metabolic disorder associated with renal dysfunction. Hemodialysis (HD) was initiated, with no loss, for metabolic control. The patient had atrial fibrillation during hemodialysis, clinical instability, and the need for reintubation. Micafungin 100 mg IV once a day [QD] was started on the 25^th^ day owing to persistent fever. Hemodynamics worsened on the 26^th^ day due to multifactorial shock.

**FIGURE 2:**
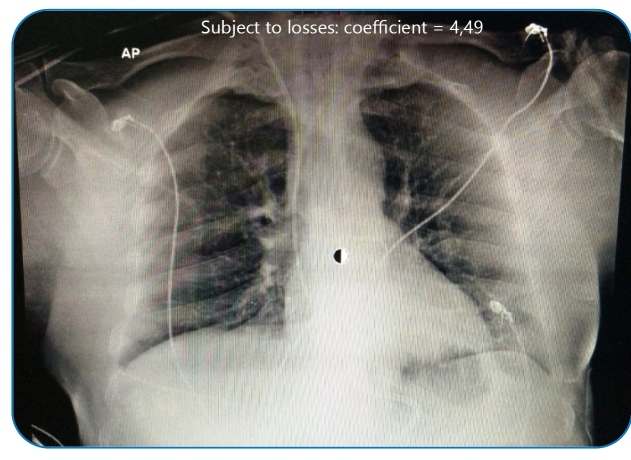
Chest radiography on the 28th day of symptoms.

Pentaglobin (a human immunoglobulin enriched in immunoglobulins, A, G and M) and pulse therapy with dexamethasone were started on the 26^th^ day (Table 1). The patient evolved within 48 h with significant clinical and laboratory improvement, extubation, and absence of fever. After 72 h, hemodialysis was discontinued. Figure 2 shows chest radiography on the 28^th^ day.

**TABLE 1:** Treatment protocol used to control the state of hyperinflammation.

Immunomodulatory drug	Route of administration	Total dose	Administration	Duration
Dexamethasone 4 mg/mL	Intravenous	40 mg (loading)	2.5 mL 6/6 h	1 day
		16 mg (maintenance)	1 mL 6/6 h	4 days
				
Pentaglobin	Intravenous	20 g	40 mL/h	3 days
50 mg/mL		(4 ampoules)		
(human immunoglobulin)				

On the 31^st^ day of symptoms, the reverse transcriptase-polymerase chain reaction (RT-PCR) test result of the nasal swab sample was negative for the novel coronavirus. Therefore, he was discharged for rehabilitation on the 48^th^ day after the initial symptoms. Approximately 8 weeks after the initial symptoms, immunoglobulin (Ig)G and IgM antibodies against SARS-CoV-2 were detected. Although the PCR test for the detection of Sars-Cov-2 on the 10^th^ day of the disease was negative, the serology with positive IgM in the 8^th^ week associated with the clinical frame confirms COVID-19.

## DISCUSSION

In severe SARS-CoV-2 infection, studies show a tendency toward macrophage activation, cytokine storm, hypercoagulability^2^, and lymphocyte apoptosis^6^. In the non-survivor group, a significant increase in interleukin-6 (IL-6) was observed^2^. Studies are still inconclusive regarding which patients tend to have a hyperinflammatory profile and at what point of disease the hyperinflammatory state will begin^7^.

Due to biological plausibility, the elderly population is susceptible to immune system dysregulation due to the process of inflammaging, in which the reserves of the stress-protection processes are reduced^8^. In epidemiological assessments of the novel coronavirus pandemic, elderly patients, as the patient in this case, are among the main risk groups in this severe form of the disease. 

Early diagnosis of MAS involves careful observation of changes in the patient's baseline clinical and laboratory status, rather than the assessment of cross-cutting criteria for hemophagocytic lymphohistiocytosis^9^, as they are sensitive in more advanced stages. Nevertheless, the increase in laboratory test results by 50% of the baseline value is sensitive information for the early diagnosis of MAS^10^, as observed on this patient’s 24^th^ day of symptoms (Table 2). Kostik et al. also showed that the combination of at least three laboratory alterations, among the nine evaluated, is sensitive for the early diagnosis of MAS in patients with systemic juvenile idiopathic arthritis (SJIA), namely: drop in albumin (< 2.9 g/dL), fibrinogen (< 1.8 g/L), platelets (< 211 × 10^9^/L), leukocytes (< 9.9 × 10^9^/L), the elevation of LDH (> 882 U/L), aspartate aminotransferase (AST > 59.7 U/L), ferritin (> 400 μg/mL), and presence of proteinuria^11^. LDH, AST, and ferritin levels are the three most consistent parameters in the literature^5^.

**TABLE 2:** Parameters evaluated for early suspicion of MAS in comparison with the reference values (Minoia et al.^10^; Kostik et al.^11^)

Parameter*	Hospital Admission	Early suspicion and	Clinical		Clinical
	Baseline	clinical worsening	instability	Immunomodulation	improvement
	10^th^ day	24^th^ day	25^th^ day	26^th^ day	28^th^ day
Platelets	211 × 10³/mm³	383 × 10³/mm³	470 × 10³/mm³	465 × 10³/mm³	167 × 10³/mm³³
Leukocytes	6.4 × 10^9^/L	**8.6** × **10** ^9^ **/L**	12.6 × 10^9^/L	24.3 × 10^9^/L	13.3 × 10^9^/L
AST	25 U/L	**78 U/L**	37 U/L	44 U/L	43 U/L
LDH	517 U/L	749 U/L	659 U/L	781 U/L	778 U/L
Ferritin	1,579 μg/L	**1,401 μg/L**	1,358 μg/L	3,339 μg/L	1,902 μg/L

*Reference values: platelets (150-450 × 10³/mm³); leukocytes (3.5-10.5 10^9^/L); AST (up to 32 U/L); LDH (< 280 U/L); ferritin (15-149 μg/L).

AST: aspartate aminotransferase; LDH: lactic dehydrogenase. **Boldfaced** numbers indicate a combination of at least three laboratory abnormalities sensitive for early diagnosis of MAS.

The standard treatment for MAS includes various immunosuppressive drugs, such as corticosteroids, calcineurin inhibitors, and anti-thymocyte globulins. Despite its use, it is still difficult to mitigate all symptoms of MAS^5^. Regarding the therapy administered to the patient, human intravenous immunoglobulin enriched in immunoglobulins, A, G and M (IVIGAM) has potential immunomodulatory properties, which has allowed its use in several reported cases of reactive MAS. The beneficial effect was established, and the time to start IVIGAM therapy was the main point of inconsistency in the reports. IVIGAM can competitively bind to the macrophage Fc receptor and alter the synthesis/release relationship of cytokines at the cellular level^12^. The initiation of IVIGAM associated with corticosteroid therapy with dexamethasone in this patient seems to have interrupted the positive feedback loop of hypercytokinemia^6^, which could lead to poor prognosis.

Early and systematic diagnosis of MAS remains a challenge because the exact cellular mechanisms of its pathophysiology are still unknown, and most clinical studies are case reports or have small cohorts with restricted populations^10^.

This case report shows the relevance of practicing early suspicion of MAS secondary to COVID-19 infection, which allowed adequate timing for the redirection of therapeutic resources toward the approach of the hyperinflammatory state.
